# Effect of Cumulative Tobacco Exposure on Blood Eosinophil Level in Chronic Obstructive Pulmonary Disease

**DOI:** 10.1155/carj/5588908

**Published:** 2025-05-22

**Authors:** İlknur Kaya, Dilek Karadoğan, Merve Yumrukuz Şenel, Tahsin Gökhan Telatar, Metin Akgün

**Affiliations:** ^1^Department of Chest Diseases, School of Medicine, Kütahya Health Sciences University, Kütahya, Turkey; ^2^Department of Chest Diseases, School of Medicine, Recep Tayyip Erdoğan University, Rize, Turkey; ^3^Department of Chest Diseases, School of Medicine, Balıkesir University, Balıkesir, Turkey; ^4^Department of Public Health, School of Medicine, Recep Tayyip Erdoğan University, Rize, Turkey; ^5^Department of Chest Disease, School of Medicine, Ağrı İbrahim Çeçen University, Ağrı, Turkey

**Keywords:** COPD, eosinophil, tobacco exposure

## Abstract

Chronic obstructive pulmonary disease (COPD) is a lung condition characterized by persistent airway obstruction and is associated with various phenotypes and endotypes. While eosinophilic inflammation is typically seen in asthma, it also occurs in COPD, with known increases in eosinophil counts during exacerbations. However, the impact of cumulative tobacco exposure on eosinophil counts is not well understood. This study aims to investigate this relationship. Data for this prospective study were collected from three centers, involving patients diagnosed with COPD. Patients' demographic data and eosinophil levels were documented. They were categorized according to GOLD Stages A, B, and E, and each group was analyzed relative to the amount of cigarette smoking. The study enrolled 227 COPD patients, predominantly male (92.5%) with an average age of 64.6 years. Of the study population, 39.8% (*n*: 90) were current smokers, and 86.9% had a smoking history of more than 20 packs/year. The average smoking history of our patients was 52.38 ± 30.69 (mean ± SD) pack/year. Our patients had an average smoking history of 39.49 ± 12.56 years. No statistically significant results were found between the amount of cigarettes smoked and eosinophil counts. However, in the correlation between smoking history and eosinophil counts, higher eosinophil counts were found in those who had former smoking compared to current smokers or never smokers. While the number of pack-years and the duration of smoking increased from Stage A to Stage E, daily cigarette consumption remained constant, and eosinophil counts did not show a significant correlation with the quantity of tobacco. Eosinophil counts in COPD patients did not vary significantly with either the amount of tobacco exposure or the severity of COPD as categorized by GOLD stages. These findings suggest that factors other than tobacco exposure may influence eosinophil levels in COPD patients.

## 1. Introduction

Chronic obstructive pulmonary disease (COPD) is a heterogeneous lung condition characterized by persistent, often progressive symptoms of chronic respiratory impairment (dyspnea, cough, and increased sputum) due to abnormalities in the airways (bronchitis and bronchiolitis) and/or alveoli (emphysema) leading to airflow obstruction [1]. The condition is also associated with numerous phenotypes and endotypes [2]. While eosinophilic inflammation is typically considered a hallmark of asthma, studies have shown that some patients with COPD, even after excluding those with asthmatic features, exhibit persistent eosinophilic inflammation. These patients respond well to corticosteroid treatment [3]. Some exacerbations in COPD are marked by increased sputum eosinophil counts, and rates of exacerbation decrease with corticosteroid treatment [4]. The GOLD guidelines mention that in cases where exacerbations are predominant, inhaled corticosteroids (ICSs) may be added based on blood eosinophil counts [1]. The effect of ICS therapy on preventing exacerbations is related to the eosinophil count in the blood. In the IMPACT study, patients previously treated with ICSs had their therapy discontinued at the start. Patients receiving only bronchodilator treatment after this discontinuation experienced more exacerbations in the first month [5].

A study by Çolak and colleagues noted that COPD patients who smoked more than 10 pack-years had higher blood eosinophil levels [6]. A national multicenter cross-sectional study compared populations diagnosed with and without COPD, finding higher eosinophil counts in the COPD population (192 cells/mL versus 160 cells/mL, *p*: 0.0003). This study also found a higher number of smokers in the COPD population compared to never smokers, and those exposed to smoke had significantly higher eosinophil counts (114 cells/mL versus 86 cells/mL, *p*: < 0.001) [7]. In this study, higher blood eosinophil counts were found in COPD patients at GOLD Stages 3 and 4 compared to Stages 1 and 2; however, the difference in cumulative tobacco exposure levels between these subgroups was not clearly examined [7]. Therefore, it is not definitively possible to state whether the eosinophilia observed in advanced-stage COPD patients is due to systemic inflammation inherent to the disease's natural progression or cumulative tobacco exposure. Most studies show an increase in airway eosinophils during exacerbations [8]. However, the role of cumulative tobacco exposure on blood eosinophil levels in COPD patients is not clearly defined.

The primary objective of this study is to examine the relationship between eosinophil levels and the degree of tobacco exposure in patients with COPD. The secondary objective is to evaluate the role of disease severity, disease duration, age, and sex on eosinophil levels.

## 2. Methods

### 2.1. Study Design

Our study is a prospective study based on data obtained with the participation of three centers. Patients were recruited from Recep Tayyip Erdogan University, Kutahya Health Sciences University, and Balikesir University. The hospitals where the study was conducted are specialized centers providing third level health services in their regions. In all three centers, diseases such as airway diseases and parenchymal diseases are treated and interventions for the diagnosis of lung cancer are performed. Data were collected between May 2022 and April 2023. According to the GOLD report, patients over the age of 40 years with appropriate clinical findings and a FEV1/FVC ratio of < 70% after reversibility were defined as COPD [1]. They were patients who came to the outpatient clinic for control purposes. There were no admissions with exacerbation of COPD. A form prepared for patients diagnosed with COPD who came to the outpatient clinic was filled out, and demographic data and hemogram values were recorded.

### 2.2. Ethics Committee Permission

Approval for our study was obtained from the Recep Tayyip Erdoğan University Non-Interventional Clinical Research Ethics Committee on May 18, 2023, with decision number 2023/122.

### 2.3. Inclusion Criteria

Patients who were admitted to the chest disease outpatient clinics of the three participating centers, who had been receiving treatment with a diagnosis of COPD for at least 1 year, and who agreed to participate in the study were included in the study. Patients over 40 years of age were included in the study. Among metabolic diseases, only diabetes mellitus and hypothyroidism were included in the study.

### 2.4. Exclusion Criteria

Patients with COPD exacerbation were not included. Patients with asthma or atopy symptoms were excluded from the study. Patients with conditions suggestive of asthma–COPD overlap syndromes, such as low smoking history or onset of symptoms at an early age, were excluded from the study. Patients diagnosed with asthma or bronchiectasis, autoimmune diseases, patients with pulmonary, hematologic, or systemic diseases affecting eosinophil levels, and patients with a history of drug use other than COPD treatment were excluded. It was ensured that they were not taking any medication that would cause eosinophilia (including anticonvulsants, allopurinol, and antibiotics such as vancomycin, minocycline, trimethoprim–sulfamethoxazole, and other sulphonamides, antituberculosis agents, and antiviral drugs).

### 2.5. Data Collection

Sociodemographic characteristics and comorbidities were recorded. To determine the degree of dyspnea, modified Medical Research Council (mMRC) and COPD Assessment Test (CAT) scores were recorded, and patients were categorized as A, B, and E according to the GOLD unified staging system in which the number of exacerbations was also used [1]. The number and percentage of eosinophils were examined among the hemogram parameters of the patients. Detailed smoking history, age at initiation of smoking, and pack/year data were recorded. The medical treatments used by the patients for respiration were recorded in detail. Pulmonary function test (PFT) parameters used to diagnose the patients were recorded and BODE indices (body mass index, airflow obstruction, dyspnea, and exercise capacity; a higher score indicates more severe disease and worse prognosis) were calculated in the light of this information.

### 2.6. Sample Size

Power analysis was used to calculate the minimum sample size to determine the relationship between blood eosinophil level and cigarette pack/year variables, which constitutes the main hypothesis of the study. The minimum sample size required for the study was determined as 207 individuals when calculated as 4 degrees of freedom with an effect size of 30% at 95% power and *α* = 0.05 level with an effect size of 30% using G-Power 3.1.9.7 software. Patients admitted to the outpatient clinic for chest diseases were included in the study until the sample size was ensured. The sampling method was not used.

### 2.7. Statistical Analysis

The statistics of the study were performed using the Statistical Package for the Social Sciences (SPSS) package program. Clinical characteristics, smoking status, pack/year information, treatments used, PFT values, and hemogram values of patients with COPD admitted to the chest diseases outpatient clinic were analyzed. The dependent variable is eosinophil count, and the independent variable is cigarette exposure and cigarette amount. Patients were categorized as A, B, and E according to GOLD staging, and each category was evaluated in comparison with the amount of smoking. Categorical variables were expressed as numbers and percentages (*n*, (%)). The chi-square test was used to compare the proportions of the groups. The relationship between eosinophil counts and pack/year quantities was analyzed by correlation analysis. Then, according to eosinophil counts (< 100 cells/mL, 100–300 cells/mL, and ≥ 300 cells/mL), age, gender, comorbidities, and cigarette pack-years were analyzed. In addition, patients were divided into 3 groups < 10 pack-years, 10–20 pack-years, and ≥ 20 pack-years according to their tobacco exposure and analyzed according to various variables. According to the multivariate regression model, it was examined whether cigarette exposure was associated with eosinophil levels independently of other variables.

## 3. Results

No statistically significant relationship was found between cumulative tobacco exposure and eosinophil count. When the relationship between tobacco exposure and eosinophil count was examined, a positive correlation was found in people who had quit smoking. There was no correlation between the GOLD stage and eosinophil count, but there was a significant difference between GOLD A and E in the eosinophil count and between GOLD B and E in eosinophil percentage (*p*: 0.002 *p*: 0.011, respectively).

### 3.1. Demographic Information of the Participants

There were 227 COPD patients in the study.  Table 1 shows the sociodemographic characteristics and smoking status of the patients according to GOLD stages. The mean age of the patients was 64.6 years (SD: 8.2). 92.5% (*n*: 210) of these patients were male. 39.8% (*n*: 90) of the study population were current smokers, and 86.9% (*n*: 187) had a smoking history of more than 20 packs/year. There was no e-cigarette use in smoking patients. The study group had the highest number of patients in Group A with 83 patients, followed by Group E with 80 patients. The majority of the study group consisted of A and E group patients (71.8%). 65.2% (*n*: 148) of the patients had comorbid diseases. Parameters according to categories are shown in Table 1.

### 3.2. Evaluation According to Tobacco Status

The mean smoking history of our patients was 52.38 ± 30.69 (mean ± SD) pack/year. Our patients had a mean smoking history of 39.49 ± 12.56 years and smoked a mean of 1.32 ± 0.65 packs per day.

When we looked at the correlation of eosinophil count with the three groups of never smokers, current smokers, and former smokers, a low-level positive correlation was found (*r*: 0.139 *p*: 0.041). The reason for this correlation was evaluated with the Kruskal–Wallis test, and eosinophil levels were found to be higher in those who were former smokers compared to the others (*p*: 0.030).

No correlation was found between the total number of packs/years, the total number of years of cigarette exposure, the number of cigarettes smoked per day, and eosinophil counts. There was also no correlation between eosinophil counts and ICS use and ICS dose.

### 3.3. Assessment According to GOLD Staging

According to the GOLD stages, the number of packs/years smoked and average years smoked increased with increasing GOLD stage. While the mean pack/year (mean ± SD) was 42.4 ± 26.9 in the GOLD A group, it was 42.9 ± 26.6 and 53.8 ± 28 in the GOLD B and GOLD E groups, respectively. The mean number of years of smoking (mean ± SD) was 32.8 ± 12.8 in the GOLD A group, 34.8 ± 11 in the GOLD B group, and 39.1 ± 12.2 in the GOLD E group, respectively. However, it was not statistically significant (*p*: 0.327). The increase in the amount of cigarettes from the GOLD A group to the E group was not accompanied by a similar increase in eosinophils. The mean eosinophil count in the GOLD A group was 0.23 ± 0.15 cells/mL 2.7 ± 1.82%, and in the GOLD B group, the mean eosinophil count was 0.25 ± 0.17 cells/mL 2.9 ± 2.2%. The highest smoking history was found in the GOLD E group (0.19 ± 0.23 cells/mL 2.19 ± 2.46%), while the eosinophil count increased slightly from GOLD A to B and was lowest in the GOLD E group.

### 3.4. Evaluation of Patients' Characteristics According to Eosinophil Count

In Table 2, COPD stages, pulmonary functions, BODE indexes, and COPD treatments were analyzed according to eosinophil levels.

**Figure 1:**
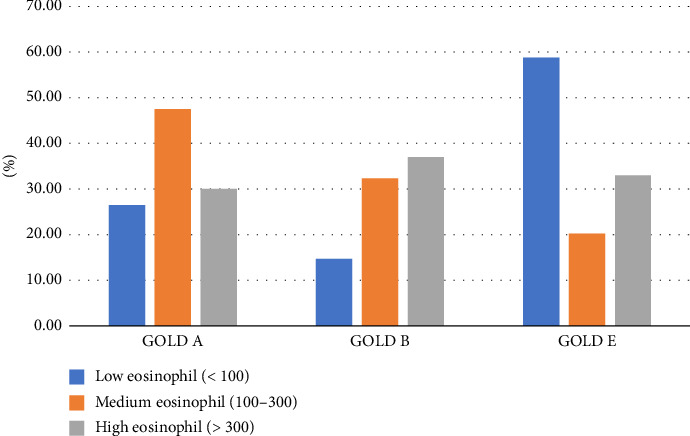
Eosinophil levels (cells/mL) according to GOLD categories.

The number of patients with eosinophil count < 100 was 29.9% (*n*: 68) and most of this group consisted of patients from the GOLD E group. In 50% of patients in the GOLD E group, eosinophil count was < 100 cells/mL. The group with eosinophil counts > 300 comprised 26.43% of the total number of patients, and the number of patients in this group was similar in all three stages (Figure 1).

It was found that the eosinophil count did not decrease gradually as the GOLD stage increased, but the mean eosinophil count and percentage were lowest in Group E. There was a significant difference between GOLD A and E in eosinophil count and between GOLD B and E in eosinophil percentage (*p*: 0.002 *p*: 0.011, respectively).

### 3.5. Evaluation of Other Factors Affecting the Level of Eosinophils

The effects of BODE index, duration of diagnosis, ICS use, and package year on eosinophil levels were evaluated by linear regression modeling, controlling for age and gender (Table 3).

Only the duration of diagnosis variable affected eosinophil levels in the model (*p*: 0.048); 16% of the change in eosinophil levels was explained by the change in the duration of diagnosis. And it is positively correlated in the same direction.

### 3.6. ICS Use According to the GOLD Stage

The rate of ICS use alone or in combination was 61.4% (*n*: 137). Most patients who did not use ICSs were in the GOLD A category. The majority (40%) of patients who used ICS alone or in combination were in the GOLD E group. In GOLD E, 28.5% (*n*: 22) of patients did not use ICS, and the difference was statistically significant (*p*: 0.003).

While 7.6% of patients received no long-term treatment, 45.2% received an ICS + LABA + LAMA combination, and the majority of these patients were in the GOLD E group (*p*: 000).

## 4. Discussion

With this study, COPD patients were examined according to demographic and clinical characteristics and compared according to the new GOLD stages. We examined whether eosinophil levels were associated with smoking exposure. Eosinophil levels were higher in patients who had former smoking compared to never smokers or current smokers. As the GOLD stage increased, the amount of cigarette use of the patients increased, although not statistically significant. However, the eosinophil count did not increase at the same rate. Approximately half of the patients in Group E had eosinophil counts < 100. However, eosinophil counts were between 100 and 300 in most patients in Groups A and B. The majority of the patients were active or former smokers. In all groups, the majority of the patients smoked more than 20 packs/year.

There are various conditions affecting blood eosinophil levels. In addition, eosinophil count is taken into consideration in the treatment phase in the GOLD guideline [1]. There are biologic agents currently used in the treatment of asthma [9]. In the GOLD 2025 report, biological agents have started to be included in the treatment of COPD. We find our study important in terms of guiding new studies.

There are various factors affecting the eosinophil count. Among these, there are various studies in which cigarette exposure has been shown. In a study examining 136 variables investigating the change in cytokine response, it was found that smoking contributed to this change and had effects comparable in magnitude with age, gender, and genetics [10]. In a study using an animal model of house dust mite–induced asthma, cigarette smoke was found to increase the number of eosinophils, interleukin-5 (IL-5), IL-13, and transforming growth factor-β in bronchoalveolar lavage fluid [11].

The predominant inflammation in COPD is neutrophilic [12], but 20%–40% is eosinophilic inflammation [13]. Recent studies have proven that tobacco exposure triggers eosinophilic inflammation in the etiopathogenesis of asthma [14]. Cigarette smoke increases eosinophil numbers through various inflammatory mechanisms associated with IL-5, a key cytokine that promotes the activation, differentiation, and survival of eosinophils. IL-5 is a critical cytokine in the regulation of the biology of eosinophils. It is produced mainly by Th2 cells, mast cells, and other immune cells and plays an important role in allergic reactions and inflammation [15]. Cigarette smoke initiates an inflammatory chain reaction in the airways, in which several immune cells such as dendritic cells, macrophages, and Th2 cells are activated. These cells secrete proinflammatory cytokines, including IL-5, which allows eosinophils to differentiate from bone marrow, survive, and accumulate in tissues [15]. In one study, an association between eosinophilic airway inflammation and even 10 packs/year or more of former cigarette smoking in a severe asthmatic population was reported [16]. These studies suggest a relationship between tobacco exposure and eosinophilia. In a recently published study, patients with COPD and severe asthma were followed up for 3 years, and eosinophil variability was examined. During this period, the eosinophil count was consistently high in the COPD group, and although not statistically significant, the number of cigarette packs/year was found to be higher in the COPD group compared to the other groups [17]. In another study, patients with and without a diagnosis of COPD were compared, and it was observed that patients with a diagnosis of COPD had a higher eosinophil count > 100. If the eosinophil count was ≤ 100, it was observed that the normal population was higher. In addition, in this study, higher blood eosinophil values were found in males, smokers, patients with a diagnosis of advanced COPD, and those with a previous diagnosis of asthma [7]. Considering this study, it may be considered that there is a relationship between tobacco exposure and eosinophil values because eosinophil counts were found to be higher in smokers. After all these studies, the eosinophilic pattern in COPD is associated with smoking. In our study, it is important that eosinophil counts were examined according to the stages of patients diagnosed with COPD, and there were no data with pack/year.

In a study, an increase in blood eosinophil levels was found in individuals without COPD to GOLD Stage III/IV COPD, and similarly, a significant negative correlation was found between eosinophils and FEV1 (%), indicating higher levels in more severe disease [7]. But in a study conducted with SPIROMICS participants who had a smoking history of more than 20 packs/year, blood eosinophils and sputum eosinophils were analyzed. Patients with sputum eosinophil counts higher than 1.25% had decreased FEV1 levels and were associated with exacerbations treated with corticosteroids. Grouping participants based only on blood eosinophils did not show any clinical differences or exacerbations, but grouping them based on sputum eosinophils revealed more distinct characteristics and was linked to COPD exacerbations [18]. Furthermore, we did not find a relationship between eosinophil count and FEV1 in our study.

In a subset of 136 patients from the SPIROMICS study, sputum samples were obtained from patients first, and then, blood and bronchoscopic lavage samples were taken 2–4 weeks later. When the patients were grouped according to smoking status and COPD disease status, no difference was found between the percentages of eosinophils in peripheral blood or sputum. However, bronchoscopic lavage eosinophils were significantly increased in active smokers compared to other groups [19]. This study found no relationship between COPD status and blood eosinophils, but it did not provide clear information about COPD stages. Our study is important in terms of providing information about COPD stages and eosinophil counts. In our study, an increase in smoking history was found in COPD patients as the stage increased, but no correlated increase was found in eosinophil counts as the stage increased.

In a study in which patients with asthma, COPD, and control groups were included and blood eosinophil counts were examined, significantly higher blood eosinophil counts were found in asthmatic individuals compared to the control group, but no difference was found between patients with COPD and control groups. Among the control group and asthmatic individuals, eosinophil counts were found to be higher in active and former smokers compared to never smokers, but no such relationship was found between eosinophil counts and smoking status in individuals with COPD [20]. In a study conducted with samples taken from 11,042 randomly selected individuals in Austria, although current smoking was compatible with high blood eosinophil counts, no association was found between blood eosinophil counts and high cumulative cigarette exposure (> 20 pack/year) [21]. There was no significant difference between the number of cigarette packs/year history in the group with persistently high and persistently low blood eosinophil counts at annual visits, whereas the number of current smokers was significantly higher in the group with persistently low eosinophil counts [13]. In a study examining the factors affecting the stability of blood eosinophil count in COPD patients, it was mentioned that there are studies that say that current smoking increases the blood eosinophil count, as well as studies that argue that it does not change. It was stated that smoking remains uncertain in this regard [22]. In our study, similar to this study, we did not find a relationship between cumulative cigarette exposure and blood eosinophilia levels. However, we found higher eosinophil levels in smokers who had former smoking compared to never smokers or current smokers.

In a study, higher eosinophil values were found in infants and young people and the male gender compared to females. Several factors including smoking, asthma, COPD, obesity, and metabolic syndrome were independently associated with high eosinophil counts [21]. In our study, BMI and mean age were similar in all 3 groups. The study population was not evaluated in terms of metabolic syndrome. This may be the reason why no association was found between eosinophil count and cumulative cigarette exposure.

Our study is valuable because it is a multicenter study. It is an important study because of obtaining detailed smoking data related to the relationship between eosinophil count and smoking. In addition to its strengths, our study also has various limitations; COPD patients are predominantly in Groups A and E according to GOLD, and there are not many patients from Group B, which may not fully represent the COPD universe. Since our study was a cross-sectional study, we could not determine whether the time of exacerbations affected the eosinophil count since it was found that the eosinophil count increased with exacerbations. Passive cigarette smoke exposure was not questioned in our patients. Ethics related to passive smoking exposure could not be evaluated. This may have led to selection bias and limitations for patients who were considered never smokers. Although we obtained valuable data despite being a three-center study, we could not reach the entire general population, which is one of our limitations. We did not evaluate the general causes of changes in eosinophil counts in patients.

Although the main inflammation in COPD is neutrophilic, it is a known fact that eosinophils are important in treatment response. The relationship between these eosinophils in the blood and smoking is a good topic for research. There is a need for studies with more patients.

## 5. Conclusion

We found that cumulative cigarette exposure had no effect on eosinophil count, which is also important in terms of response to attacks in COPD patients. However, according to our study, in COPD patients who were former smoking, current smokers, and never smokers, we found higher eosinophil levels in the former smoking group. We found that cigarette exposure is one of the factors that may affect the eosinophil count, and we think that this study is important because it is a study that can guide the treatment in patients with COPD.

## Figures and Tables

**Table 1:** Demographic characteristics of patients according to GOLD categories.

	Total 100% (*n*: 227)	A (36.6%) (*n*: 83)	B (28.2%) (*n*: 64)	E (35.2%) (*n*: 80)	*p*
Age median (IQR)	66 (10)	65 (12)	66 (9)	66 (11.50)	0.182
Age/mean ± SD	64.6 ± 8.2	63.15 ± 7.63	65.76 ± 7.04	65.40 ± 9.49	
BMI, kg/m^2^ median, (IQR)	25.71 (6.77)	25.73 (6.81)	27.34 (7.63)	24.44 (5.42)	0.357
BMI, kg/m^2^ mean ± SD	25.94 ± 4.6	25.94 ± 4.04	26.41 ± 4.98	25.55 ± 4.89	
Gender (*n*, %)					0.269
Female	17 (7.5%)	4 (4.8%)	4 (6.3%)	9 (11.3%)	
Male	210 (92.5%)	79 (95.2%)	60 (93.8%)	71 (88.8%)	
Comorbid disease presence					0.815
Absent	79 (34.8%)	28 (33.7%)	21 (32.8%)	30 (37.5%)	
Present	148 (65.2%)	55 (66.3%)	43 (67.2%)	50 (62.5%)	
Metabolic diseases	60 (36.36%)	21 (35%)	17 (36.95%)	22 (37.28%)	0.853
Cardiovascular diseases	93 (56.36%)	36 (60%)	25 (54.34%)	32 (54.23%)	0.637
Malignancy	12 (7.27%)	3 (5%)	4 (8.69%)	5 (8.47%)	0.736
Smoking status					0.611
Never	14 (6.2%)	5 (6.0%)	6 (9.4%)	3 (3.8%)	
Former smoker	122 (53.7%)	42 (50.6%)	34 (53.1%)	46 (57.5%)	
Current smoker	91 (40.1%)	36 (43.4%)	24 (37.5%)	31 (38.7%)	
Smoking amount (pack/year)					0.472
< 10	10 (4.7%)	3 (3.8%)	4 (6.8%)	3 (3.9%)	
10–20	19 (8.8%)	10 (12.7%)	5 (8.5%)	4 (5.2%)	
20 and over	186 (86.5%)	66 (83.5%)	50 (84.7%)	70 (90.9%)	
Smoking initiation age					0.327
< 18	12 (56.3%)	41 (52.6%)	31 (52.5%)	48 (63.2%)	
18 and over	93 (43.7%)	37 (47.4%)	28 (47.5%)	28 (36.8%)	
Presence of family history of respiratory diseases					0.649
Present	46 (21.4%)	14 (17.9%)	14 (23.3%)	18 (23.4%)	
Absent	169 (78.6%)	64 (82.1%)	46 (76.7%)	59 (76.6%)	
Long-acting inhaler types					< 0.01^∗^
Only LAMA	20 (8.96%)	6 (7.22%)	9 (14.06%)	5 (6.57%)	
Only ICS	3 (1.34%)	0	0	3 (3.94%)	
LABA + LAMA	49 (21.97%)	32 (38.55%)	10 (15.62%)	7 (9.21%)	
LABA + ICS	33 (14.79%)	13 (15.66%)	9 (14.06%)	11 (14.47%)	
LABA + LAMA + ICS	101 (45.29%)	26 (31.32%)	33 (51.56%)	42 (55.26%)	
None	17 (7.62%)	6 (7.22%)	3 (4.68%)	8 (10.52%)	
Spirometry mean ± SD					< 0.01^∗^
FEV1 (L)	1.50 ± 0.60	1.78 ± 0.57	1.44 ± 0.56	1.14 ± 0.49	
FEV1%	50.8 ± 18.17				
FEV1/FVC	57.04 ± 9.83	60.18 ± 7.99	59.67 ± 37	51.58 ± 9.83	

*Note: n*, sample size.

Abbreviations: BMI, body mass index; ICS, inhale corticosteroid; IQR, interquartile range; LABA, long-acting beta-2 agonist; LAMA, long-acting muscarinic antagonist.

^∗^Kruskal–Wallis test.

**Table 2:** Characteristics of the patients according to eosinophil levels.

	Total sample (*n*: 227)	Eosinophil levels			*p*
		< 100 cells/mL (*n*: 62)	100–300 cells/mL (*n*: 98)	> 300 cells/mL (*n*: 56)	
Age median (IQR)	66 (10)	66.5 (11.3)	64 (9.5)	66 (7.8)	0.530
BMI, kg/m^2^ median, (IQR)	25.7 (6.8)	24.1 (6.3)	26.3 (5.9)	25.7 (6.3)	0.229
Gender (*n*, %)					0.400
Female	17 (7.9%)	5 (8.1%)	10 (10.2%)	2 (3.6%)	
Male	199 (92.1%)	57 (91.9%)	88 (89.8%)	54 (96.4%)	
Comorbid disease presence					0.144
Absent	74 (34.3%)	27 (43.5%)	32 (32.7%)	15 (26.8%)	
Present	142 (65.7%)	35 (56.5%)	66 (67.3%)	41 (73.2%)	
Smoking status					0.083
Never	13 (6.0%)	2 (3.2%)	6 (6.1%)	5 (8.9%)	
Former smoker	118 (55.2%)	43 (69.4%)	50 (51.0%)	25 (44.6%)	
Current smoker	85 (39.8%)	17 (27.4%)	42 (42.9%)	26 (46.4%)	
Smoking amount (pack/year)					0.329
< 10	9 (4.4%)	2 (3.3%)	2 (2.2%)	5 (9.6%)	
10–20	18 (8.8%)	6 (9.8%)	7 (7.6%)	5 (9.6%)	
20 and over	178 (86.8%)	53 (86.9%)	83 (90.2%)	42 (80.8%)	
Smoking initiation age					0.704
< 18	118 (58.1%)	37 (61.7%)	53 (58.2%)	28 (53.8%)	
18 and over	85 (41.9%)	23 (38.3%)	38 (41.8%)	24 (46.2%)	
Inhaler device number					0.008^∗^
Single device	71 (34.1%)	14 (24.6%)	44 (44.9%)	13 (24.5%)	
More than one device	137 (65.9%)	43 (75.4%)	54 (55.1%)	40 (75.5%)	
ICS use					0.790
Present	133 (62.4%)	40 (65.6%)	59 (60.2%)	34 (63.0%)	
Absent	80 (37.6%)	21 (34.4%)	39 (39.8%)	20 (37.0%)	
ICS dose					0.410
Medium	22 (16.8%)	9 (23.1%)	9 (15.5%)	4 (11.8%)	
High	109 (83.2%)	30 (76.9%)	49 (84.5%)	30 (88.2%)	
Only SABD user					0.717
Present	13 (6.1%)	5 (8.2%)	5 (5.1%)	3 (5.6%)	
Absent	200 (93.9%)	56 (91.8%)	93 (94.9%)	51 (94.4%)	
Long-acting inhaler types					0.314
Only LAMA	20 (9.0%)	7 (10.4%)	9 (9.2%)	4 (6.9%)	
Only ICS	3 (1.3%)	3 (4.5%)	0 (0.0%)	0 (0.0%)	
LABA + LAMA	49 (22%)	12 (17.9%)	26 (26.5%)	11 (19%)	
LABA + ICS	33 (14.8%)	11 (16.4%)	12 (12.2%)	10 (17.2%)	
LABA + LAMA + ICS	101 (45.3%)	27 (40.3%)	45 (45.9%)	29 (50%)	
None	17 (7.6%)	7 (10.4.5%)	6 (6.1%)	4 (6.9%)	
Spirometry mean ± SD					
FEV1 (L)	1.50 ± 0.61	1.39 ± 0.57	1.55 ± 0.61	1.53 ± 0.63	0.303
FEV1%	50.82 ± 18.17	48.42 ± 18.80	51.42 ± 17.04	52.58 ± 19.58	0.474
FEV1/FVC	57.04 ± 9.83	60.34 ± 15.24	66.52 ± 13.89	63.5 ± 14.01	0.073
BODE mean ± SD	3.47 ± 2.58	4.64 ± 2.82	3.08 ± 2.52	3.19 ± 2	0.004^∗^

*Note: n*, sample size.

Abbreviations: BMI, body mass index; ICS, inhale corticosteroid; IQR, interquartile range; LABA, long-acting beta-2 agonist; LAMA, long-acting muscarinic antagonist.

^∗^Kruskal–Wallis test.

**Table 3:** Other factors that may affect eosinophil levels.

	Standardized coefficients beta	*t*	Lower bound	Upper bound	*pvalue*
BODE index	−0.015	−0.185	−32.306	26.766	0.853
Diagnosis period	0.160	1.994	0.129	25.834	0.048^∗^
ICS users	−0.093	−1.140	−252.177	67.599	0.256
Package (years)	−0.056	−0.716	−4.116	1.925	0.475

Abbreviation: ICS, inhale corticosteroid.

^∗^The linear regression model was adjusted for age and sex.

## Data Availability

The data that support the findings of this study are available from the corresponding author upon reasonable request.
